# Observations on Measles

**Published:** 1816-07

**Authors:** Henry Ronalds


*For the London Medical and Physical Journal.*

### *Observations on Measles;* by HENRY RONALDS, M.D.

"A considérer les maladies par leurs causes, ou par leurs circonstances déterminantes, et par la liason, les rapports et la gravité de leurs symptomes; c'est à dire, à les considérer dans leur ensemble, et sous tous leurs points de vue, l'une ne resemble jamais à l'autre. Deux rhumes, deux simples fièvres éphémères ne sauroient etre exactement les memes: il y a toujours comme dans les physionomies les plus semblables en apparence, des traits, ou des nuances qui les distinguent. Or les moindres modifications dans leur charactère, devant en apporter d'analogues dans leur traitement, il importe d'étudier chaque cas en lui-meme, afin de tirer de la combinaison, ou de la dépendance naturelle de ses diverses phénomènes un plan raisonné de conduite."

CABANIS.

I TAKE up the pen with the hope of inducing some of your experienced correspondents to favour the profession with an account of their practice in measles, a disease which, from the formidable symptoms it occasionally assumes, well merits, in my opinion, a closer investigation.

The disease is at present epidemic in our neighbourhood, and has chiefly attacked the children of the poor, among whom it has committed great ravages. In its milder form it commences by drowsiness, watery eyes, sneezing, a short dry cough nearly characterizing the disorder by its peculiarity, and a wheezing respiration; the pulse is quick, and rather full; the face becomes flushed; a diarrhœa generally comes on early, and accompanies the farther progress. About the fifth day the eruption makes its appearance, when the pulmonary symptoms are alleviated, but the synocha does not appear to subside: in most instances it is rather increased. After a few days the eruption ends, as usual, in a branny desquamation, and the patient very gradually recovers.

Such is a sketch of the progress of the more benign species. Concerning the malignant kind, which is my present object, I find the earliest writer who has given an accurate description to be Sydenham, and here allow me to refer to his 4th sect. and 3d chapter. The disease commences with the usual symptoms; but there is nothing like certainty in the appearance of the rash. In one case it broke out on the fourth day, in others not till the eleventh or twelfth; and, from the commencement of indisposition, the pulmonary disorder became uncontroulable. I may venture to say that in this malignant species the eruption was always very inconsiderable, and on the face could scarcely be at all perceived. In confirmation of Sydenham's observation, I remarked that it began on the shoulders and breast, gradually extending downwards to the thighs and hips.

CASE.—I was desired, on the 8th of May, to visit a little girl, two years of age, in whom, about five days ago, the usual symptoms manifested themselves. I found her greatly distressed with dyspnœa, a short dry cough, disordered bowels, the stools consisting of a dark offensive matter. Other symptoms of debility and putrescency were not wanting, as inability to speak or cry, brown and furred tongue, sordid collections between the teeth, pallid countenance, approaching to livid. With the hope of bringing out the eruption, warm bathing was immediately had recourse to; a blister was likewise applied to the chest, but both methods failed in producing the desired effect. The only good consequence of the blister was the presence of a slight expectoration. In this case, and others resembling it, I was fearful of using general bleeding, or even leeches to the chest, as a sudden suppression of the rash might be in danger of aggravating the pulmonary complaints. The infancy of the patient precluded the use of bark and acid; wine was, however, after some difficulty, administered. The medicines had recourse to were the Mist. Amygd. with Potass. Nitras. The patient was kept in a moderately warm room. Notwithstanding all our efforts, the septic tendency became more manifest; and, had not the epidemic been very general throughout the town, this disease could scarcely have been denominated measles, as only a slight efflorescence appeared on the tenth day, first on the shoulder, and afterwards on one thigh and leg, the face not being perceptibly invaded.

This case, with one other during my practice in this epidemic, terminated fatally, and I am thus inclined to publish their history, as I have been taught that measles, under proper treatment, is not to be considered a dangerous disease. Dr. Gregory says,* that, in the course of thirty-two years

* Prælect. Gregor. M.S.

practice, he has not lost a single patient in the disease; but he takes occasion to inform his pupils, that a malignant species has begun to appear in Scotland.

We are informed that at Leith, during one epidemic, not less than 250 patients were carried off, notwithstanding the acknowledged abilities of Dr. Kellie and other respectable practitioners resident there.

But, to proceed farther back, we shall find the existence of Rubeola maligna fully established, not only by Sydenham, as I have elsewhere observed, but by Morton, who observes that he lost 300 patients in one week, of the putrid measles, in London.*

On the whole, the non-appearance of this intractable complaint, during the many years' practice of Dr. Gregory at Edinburgh, serves to shew that it is a very rare phenomenon. A very variable atmosphere is most probably the remote cause, as all catarrhal and pneumonic affections are peculiarly apt to prevail when the weather is in an unsettled state; and it is to be observed, that the late winter and spring have been remarkable for sudden changes of temperature.

The disease in this place has commonly, and not improperly, obtained the appellation of †*black* measles, expressing at once a horror at its fatality, and a peculiar dark or livid countenance in the patient. This latter symptom, which usually preceded the fatal moment, is most probably connected with a considerable effusion into the cellular texture of the lungs; but I am sorry that no opportunity, in these two unfortunate cases, presented itself of verifying this opinion by dissection.

It is proved, from a great number of dissections of those who have died of exanthematous diseases, that the eruption has attacked the stomach and whole alimentary canal. In the small-pox, a very characteristic symptom is pain at the pit of the stomach, and it is now generally considered that

* These facts, I think, set at rest all conjectures of measles being rendered more severe by the prevalence of vaccination. For further confirmation, see a paper in Medico-Chirurg. Trans. vol. v. p. 441, in which it appears that the number of deaths from measles after small-pox is as 1 in 19; after cow-pox as 1 in 29.

† This appearance, however, differs from that described by the scientific Willan: he divides Rubeola into three species, viz. the Rubeola vulgaris, Rubeola sine catarrho, and R. nigra; in which latter species the papulæ suddenly assume a black or dark purple colour. The same excellent physician has established the fact of the recurrence of the disease; but it is confined to those patients who have had it without the catarrhal symptoms.

the the uneasy sensation is to be attributed to small-pox pustules. From this analogy, we may be safe in concluding that the mucous membrane of the larynx, trachea, and bronchiæ are invaded with the eruption in measles; and may it not be inferred that the danger is greater in proportion to the energy of the system not proving sufficient to throw out the eruption to the surface? In scarlatina, the debility, and, consequently, the danger, become greater, as the throat and fauces are violently affected.

The most prominent features in the disorder which I have attempted to describe are the existence of typhoid symptoms along with a severe pneumonic affection: in this the epidemic differs from that described by many writers who have witnessed the presence of typhus, and, consequently, putrescence, after the disappearance of the rash;* it more nearly resembled one which prevailed in Edinburgh during the winter of 1808-9. The following remarks, by a judicious critic, I shall venture to transcribe.

"By† almost every writer, the measles has been considered as a highly inflammatory disease, demanding the strictest antiphlogistic treatment, and, according to the violence of the pneumonic symptoms, the free use of the lancet. The epidemic which raged here last winter taught us the danger of this indiscriminate practice. The great depression of the vis vitæ, which was so remarkable in this measles, the frequent livid appearance of the eruption, the tendency of the blistered parts to ulceration and gangrene, the actual occurrence of gangrene in some cases, the failure of evacuations in relieving the alarming symptoms of peripneumony, the increased difficulty of breathing, and the great sinking which followed the application of even a few leeches, led us at last to be very sparing in every evacuation, except purging, and in many cases to have recourse to wine, bark, and digitalis. And we had every reason to congratulate ourselves in our change of practice."

Dr. Home has proposed and practised inoculation in measles. He found, in the few cases on whom the experiment was made, that the disease became considerably milder. The operation consisted in drawing a drop of blood with a lancet near to one of the papulæ, imbuing a thread of cotton, and inserting this under the cuticle of the person to be inoculated. The plan has been adopted, since his time, upon a

* Vide Watson, Lond. Medical Observ. vol. iv. Also Bateman, Ed. Med. Journal, vol. viii. p. 514.

† Edin. Med. Journal, vol. v. p. 366.

small small scale, apparently without furnishing the expected results, but it certainly appears deserving of a more extended trial.

*Brentford;*

*May 22d,* 1816.

